# *Mycoplasma tracheobuteonis* sp. nov., a Novel Respiratory *Mycoplasma* Species from the Common Buzzard (*Buteo buteo*)

**DOI:** 10.3390/microorganisms14061224

**Published:** 2026-05-29

**Authors:** Sarah Kugler, Anna Kübber-Heiss, Nora Dinhopl, Angelika Auer, Igor Loncaric, Volker Schmidt, Ana S. Ramirez, Joachim Spergser

**Affiliations:** 1 Research Institute of Wildlife Ecology, Department of Interdisciplinary Sciences, University of Veterinary Medicine, 1160 Vienna, Austria; 2 Unit Pathology, Department of Biological Sciences and Pathobiology, University of Veterinary Medicine, 1210 Vienna, Austria; 3 Unit Infectiology and Virology, Department of Biological Sciences and Pathobiology, University of Veterinary Medicine, 1210 Vienna, Austria; 4 Unit Microbiology, Department of Biological Sciences and Pathobiology, University of Veterinary Medicine, 1210 Vienna, Austria; 5 Clinic for Birds and Reptiles, Faculty of Veterinary Medicine, University of Leipzig, 04103 Leipzig, Germany; 6 Instituto Universitario de Sanidad Animal y Seguridad Alimentaria, University of Las Palmas de Gran Canaria, 35413 Arucas, Spain

**Keywords:** novel mycoplasmas, respiratory tract, common buzzard

## Abstract

Mycoplasmas are frequently recovered from the upper respiratory tract of birds of prey, yet many isolates remain taxonomically unresolved. In the present study, a collection of ten previously unclassified *Mycoplasma* strains, predominantly isolated from the trachea of the common buzzard (*Buteo buteo*), was subjected to comprehensive phenotypic and genomic characterization. All strains grew well in modified Hayflick’s medium and formed colonies with the characteristic fried-egg appearance. None of the strains produced acid from glucose or hydrolyzed arginine or urea. Phylogenetic analyses based on 16S rRNA gene, 16S–23S intergenic spacer, and partial *rpoB* gene sequences placed the strains within the *Mycoplasma synoviae* cluster, in close proximity to five recently described *Mycoplasma* species associated with raptors such as eagles and kites. Matrix-assisted laser desorption ionization–time of flight (MALDI-ToF) mass spectrometry enabled the clear discrimination of the investigated strains from closely related taxa. Whole-genome comparisons, together with phylogenomic analyses, supported the assignment of these strains to a novel species within the genus *Mycoplasma*. The name *Mycoplasma tracheobuteonis* sp. nov. is proposed, corresponding to its preference for colonizing the upper respiratory tract of the common buzzard, with strain 48589B^T^ (=DSM 115882^T^ = NCTC 14927^T^) designated as the type strain.

## 1. Introduction

Members of the class *Mollicutes* are small prokaryotes characterized by the absence of a cell wall, a minute cellular and genomic size, and a low genomic G+C content. The genus *Mycoplasma* (commonly referred to as mycoplasmas) represents the most diverse lineage within the class *Mollicutes* and currently comprises more than 130 recognized species. They colonize a wide range of hosts, including humans and other mammals as well as birds, reptiles, fish, and mollusks. Mycoplasmas generally exhibit pronounced host and tissue specificity, predominantly inhabiting the respiratory and urogenital mucosa, but also ocular, mammary and joint tissues, as well as the blood. Several species are considered commensals, whereas others are well recognized as opportunistic or primary pathogens [1,2].

In birds, mycoplasmas have been studied predominantly in domestic poultry and waterfowl, whereas their diversity in birds of prey remains less well characterized [3,4]. Predatory birds are considered frequent carriers, particularly within *Falconiformes* [5,6,7], but comparatively little is known about *Mycoplasma* species associated with the family *Accipitridae* (order *Accipitriformes*), which includes eagles, hawks, buzzards, kites, harriers, and Old World vultures.

Only a limited number of species are regarded as primarily associated with accipitrid hosts, including *Mycoplasma* (*M*.) *buteonis*, *M. gypis*, and *M. neophronis*, reported mainly from common buzzards, Eurasian griffon vultures, and Canarian Egyptian vultures, respectively [8,9]. A non-cultivable organism provisionally designated “*Mycoplasma vulturii*” has also been detected in Oriental white-backed vultures [10]. Additional mycoplasmas recovered from raptors include *M. falconis*, *M. hafezii*, and *M. seminis*, primarily in falcons but occasionally in *Accipitridae* [5,6,7,11,12], and *M. corogypsi* in black vultures [13,14,15]. Molecular investigations have further indicated the presence of additional undescribed *Mycoplasma* species in predatory birds, highlighting the incomplete understanding of their diversity and host associations [16,17,18,19].

Recently, we described five novel *Mycoplasma* species—*M. aquilae*, *M. paraquilae*, *M. milvi*, *M. haliaeeti*, and *M. razini*—isolated from the respiratory tract of eagles and kites, thereby expanding the recognized diversity of accipitrid-associated mycoplasmas [20]. The organism described in the present study is phylogenetically closely related to this group, sharing 98.75–99.10% 16S rRNA gene sequence similarity with these taxa, but it can be clearly differentiated by MALDI-ToF mass spectrometry [21]. Moreover, it was recovered predominantly from the trachea of the common buzzard (*Buteo buteo*), suggesting a distinct taxonomic entity with apparent host association.

In the present study, eight isolates obtained from the respiratory tract of the common buzzard and two from additional raptors were subjected to comprehensive phenotypic and genetic characterization in accordance with guidelines and recommendations for the description of new species in the class *Mollicutes* [22], supplemented by whole-genome-based analyses. Comparative analyses, including MALDI-ToF mass spectrometry, phylogenetic reconstruction, and genomic relatedness indices, demonstrated that these strains represent a coherent and previously undescribed taxon within the genus *Mycoplasma*. Based on the data presented, the name *Mycoplasma tracheobuteonis* sp. nov. is proposed for this organism, given that it has been predominantly isolated from the trachea of the common buzzard.

## 2. Materials and Methods

### 2.1. Primary Isolation, Cultural and Morphological Characterization

Samples from *Accipitridae* were taken during necropsies at the Research Institute of Wildlife Ecology, University of Veterinary Medicine, Vienna, Austria as well as during field investigations of raptors conducted in Germany and Spain. Most birds were healthy individuals that died from non-infectious causes (e.g., trauma, poisoning, or emaciation), and no relevant gross or histologic respiratory lesions were identified. Tracheal or choanal swabs were placed into Amies transport medium and stored at 4 °C until processing at the Microbiology diagnostic unit at the University of Veterinary Medicine Vienna, Austria. For mycoplasma isolation, swabs were suspended in 1 mL of 2SP medium (containing 0.2 mol/L sucrose in 0.02 mol/L phosphate buffer (pH 7.0 ± 0.2) composed of mono- and dibasic phosphate salts and supplemented with 10% fetal calf serum) and mixed by vortexing for 30 s. Aliquots of 100 µL were inoculated onto modified Hayflick’s agar [23] and incubated at 37 °C in an atmosphere containing 5% CO_2_ for a maximum of 7 days. Individual colonies displaying mycoplasma-like morphology were subsequently transferred into 5 mL modified Hayflick’s broth [23] and cultivated at 37 °C under ambient atmospheric conditions for 3 to 4 days. Colony-derived cultures from 2016 and earlier were identified using 16S rRNA gene sequencing followed by phylogenetic analysis (see below). From 2017 on, colony-derived cultures were identified by MALDI-ToF mass spectrometry as described previously [21]. Unclassified *Mycoplasma* isolates were maintained at −80 °C until additional investigations were performed. For the present study, ten representative strains were selected from a group (n = 36) of unknown but closely related *Mycoplasma* isolates, which were predominantly obtained from the common buzzard and exhibited close phylogenetic relationships to the species of the recently described *M. aquilae* species complex and *M. razini* [20]. Selection was based on host species and country and year of isolation, with a focus on epidemiological unrelatedness (Table 1).

The ten selected strains underwent comprehensive taxonomic analysis, beginning with triple-filter cloning [24] to ensure pure cultures, and cultivation in/on modified Hayflick’s medium [23] at different temperatures (4 °C, 15 °C, room temperature, 28 °C, and 42 °C) and conditions (aerobic and anaerobic) to determine culture characteristics and colony morphology. Furthermore, cultures were filtrated through membrane filters with pore sizes of 450 and 220 nm. Microcinematography was performed to assess gliding motility as described previously [25]. In addition, the cell morphology of the proposed type strain was determined by transmission electron microscopy as described before [9].

### 2.2. Phylogenetic Analyses

DNA was extracted from pellets obtained from 9 mL broth cultures following centrifugation at 20,000× *g* for 10 min using the DNeasy Blood & Tissue Kit (Qiagen, Hilden, Germany). Nearly full-length 16S rRNA gene sequences of all ten isolates were amplified with primers 27f and 1492r [26]. To further determine the relatedness of the novel species to established *Mycoplasma* species, the 16S–23S intergenic spacer region (ISR) and a partial fragment of the *rpoB* gene were investigated according to previously published protocols [20,27]. Amplicons were purified using the Exo-CIP^TM^ Rapid PCR Cleanup Kit (New England Biolabs, Ipswich, MA, USA) and subjected to bidirectional sequencing at LGC Genomics (Berlin, Germany). Sequence similarities for the 16S rRNA gene, ISR, and partial *rpoB* gene were determined using EzBioCloud [28] and BLASTn searches against GenBank databases [29], respectively. Multiple sequence alignments of 16S rRNA and partial *rpoB* gene sequences were generated with ClustalW [30]. Phylogenetic analyses including closely related taxa were carried out in MEGA11 using the maximum likelihood method based on Tamura-Nei substitution model with 1000 bootstrap replicates [31]. Final tree visualization and annotation were performed using iTOL v6 [32].

### 2.3. Standard Phenotypic Analyses and MALDI-ToF Mass Spectrometry

The investigated strains were tested for glucose utilization, arginine and urea hydrolysis [33], hemolysis [34], and production of ‘film and spots’ [35]. MALDI-ToF mass spectrometric analyses of broth cultures derived from the ten investigated strains as well as from phylogenetically related *Mycoplasma* species were carried out as previously reported [21]. Reference spectra, also known as main spectrum profiles (MSPs), were generated from the acquired spectra. Based on an arbitrary distance matrix, a score-oriented dendrogram was calculated using the correlation distance measure in combination with the average linkage algorithm implemented in Bruker Daltonics software package (MBT Compass HT version 2025) (Bremen, Germany).

### 2.4. Genome Sequencing, Genome Coherence, and Phylogenomic Analyses

For whole-genome sequencing, all ten strains were cultivated in 20 mL modified Hayflick’s medium at 37 °C in ambient atmospheric conditions for 4 days. Cells were subsequently harvested by centrifugation at 20,000× *g* for 10 min, and genomic DNA was purified using the DNeasy Blood and Tissue Kit (Qiagen, Hilden, Germany). DNA concentration and integrity were evaluated by fluorometric quantification using Qubit^TM^ 4 fluorometer (Thermo Scientific, Waltham, MA, USA) and by capillary electrophoresis employing the Tape Station 4150 system (Agilent, Santa Clara, CA, USA). Sequencing of all strains was conducted on an Illumina MiniSeq platform (2 × 150 bp; Illumina, San Diego, CA, USA). Adaptor sequences and low-quality reads were removed with the BBDuk plugin implemented in Geneious Prime^®^ 2025.02 (Biomatters Ltd., Auckland, New Zealand). Filtered reads were assembled de novo using SPAdes 4.0.0. [36] with default parameters. The proposed type strain 48589B^T^ was additionally subjected to long-read sequencing on a MinION device (Oxford Nanopore Technologies, Oxford, UK). Libraries were prepared using the Ligation Sequencing Kit V14 (SQK-LSK114), and the sequencing run was performed on a FLO-MIN114 flow cell. Base calling was carried out with MinKNOW version 25.05.12. Generated FASTQ files were processed in Geneious Prime 2025.02, and reads shorter than 1000 bp were excluded. Hybrid genome assembly combining Illumina short reads and Nanopore long reads was performed using Unicycler version 0.5.1 with default settings [37]. The circularized complete genome of strain 48589B^T^ as well as draft genome assemblies of the remaining nine strains were annotated using the NCBI Prokaryotic Genome Annotation Pipeline (PGAP).

Genomic relatedness was assessed by calculating average nucleotide identity values based on BLAST (ANIb) and MUMmer (ANIm) algorithms as well as tetranucleotide signature correlation (TETRA) coefficients using the JSpeciesWS online software package (version 5.0.3) [38]. Digital DNA-DNA hybridization (dDDH) estimates were additionally obtained with the Genome-to-Genome Distance Calculator (GGDC, version 3.0), applying the recommended formula, formula 2, based on identities/high-scoring segment pair (HSP) length [39]. For phylogenomic analysis, a genome-based phylogenetic tree including the investigated strains and closely related *Mycoplasma* species was generated using the Codon Tree approach implemented at the Bacterial and Viral Bioinformatics Resource Center (BV-BRC). This method evaluates single-copy global protein families (PGFams) and reconstructs phylogenies using Randomized Axelerated Maximum Likelihood (RAxML, version 8.2.11) combined with fast bootstrap analysis [40]. Final visualization and annotation of the phylogenomic tree were carried out with iTOL v6 [32].

For comparative genomics, the genomes generated from the ten strains were compared by analyzing whole-genome alignments using progressive Mauve [41] implemented in Geneious Prime^®^ (Biomatters Ltd., Auckland, New Zealand). Functional genomic analyses were performed by comparing selected genomic regions and predicted protein sequences against the NCBI non-redundant protein database using BLASTp. Matches showing ≥60% query coverage and ≥40% amino acid identity were regarded as significant.

## 3. Results and Discussion

### 3.1. Cultural and Morphological Traits

**Figure 1:**
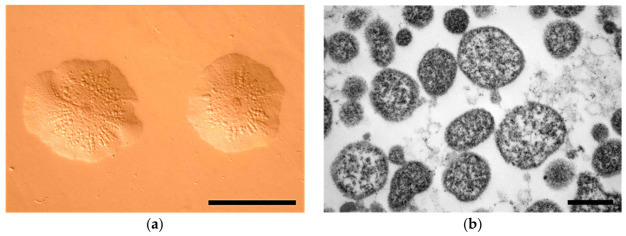
(**a**) Fried-egg colonies of 48589B^T^ on modified Hayflick’s Agar. Bar, 500 μm; (**b**) Spherical- to oval-shaped cells with a discrete fuzz-like coating; transmission electron micrograph of 48589B^T^. Bar, 500 nm.

The *Mycoplasma* strains were grown on modified Hayflick’s agar incubated at 37 °C in an atmosphere containing 5% CO_2_ for up to 10 days. Typical umbonate fried-egg colonies became visible after approximately 3 days of incubation. The colonies showed irregular margins and a radially granulated surface structure (Figure 1a). The colony diameters ranged from 200 to 800 μm. The strains did not exhibit satellite colony formation, which is indicative of gliding motility [22], a result corroborated by a microcinematographic examination. The strains showed rapid growth at 37 °C, whereas only limited growth was observed at room temperature (approximately 20–22 °C), 28 °C, and 42 °C. No colony formation occurred at 4 °C or 15 °C. Growth under anaerobic conditions at 37 °C was likewise observed using the GasPak™ system (BD Diagnostics, Franklin Lakes, NJ, USA). The isolates were capable of passing through membrane filters with pore sizes of 450 nm and 220 nm. Filtration through the 220 nm membrane resulted in an approximately 1 log_10_ cfu/mL reduction in viable colony counts. Transmission electron microscopy of the proposed type strain 48589B^T^ revealed pleomorphic cells lacking a cell wall and enclosed by a single membrane with a fuzz-like coating. The cells contained a finely granular cytoplasm with variably electron-dense regions, possibly representing ribosomes and sections of the circular chromosome (Figure 1b) [42]. The cultural and morphological characteristics of strain 48589B^T^ were indistinguishable from those previously described for other *Mycoplasma* species isolated from buzzards, including *M. buteonis* and members of the *M. aquilae* complex [8,20].

### 3.2. Phylogenetic Positioning

**Figure 2:**
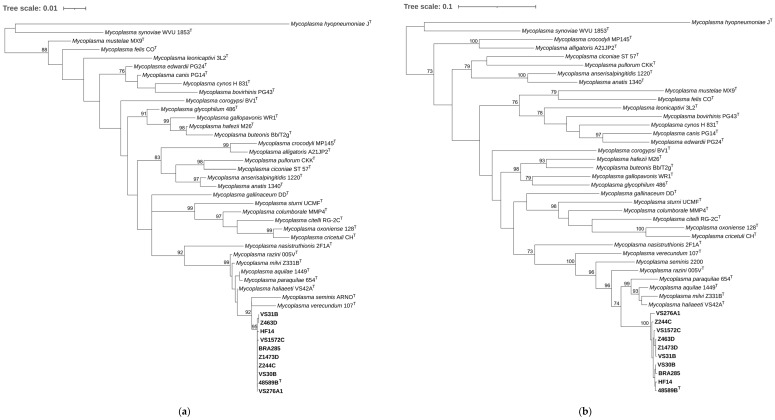
Maximum likelihood trees, demonstrating the phylogenetic relatedness of the 10 strains studied to related species of the *M. synoviae* cluster based on (**a**) 16S rRNA genes and (**b**) partial *rpoB* gene sequences. *M. hyopneumoniae* J^T^ (*M. neurolyticum* cluster) was used as out-group organism. Numbers at nodes represent bootstrap confidence values (1000 replications). Only values ≥ 70% are shown. Tree scale, number of nucleotide substitutions per site.

The comparative analysis of the 16S rRNA gene sequences assigned all ten isolates to the genus *Mycoplasma*. The nearly complete 16S rRNA gene sequence of strain 48589B^T^ (1437 nt) showed the highest similarity to accipitrid-associated *M. haliaeeti* VS42A^T^ (99.10%), *M. aquilae* 1449^T^ (98.96%), *M. razini* 005V^T^, *M. paraquilae* 654^T^ (both 98.82%), and *M. milvi* Z331B^T^ (98.75%), as well as to *M. verecundum* 107^T^ (98.66%) and *M. seminis* ARNO^T^ (98.47%). The 16S rRNA gene sequences of 48589B^T^, BRA285, VS30B, VS276A1, Z244C, and Z1473D were identical and differed only slightly from those of HF14, VS31B, VS1572C, and Z463D (one base difference). Despite these high 16S rRNA gene sequence similarity values with the most closely related species, the resulting phylogenetic tree reveals a clear separation of 48589B^T^ (and related strains) from their closest relatives, a finding supported by a high bootstrap value. In summary, the phylogenetic analysis of 16S rRNA gene sequences shows that the ten strains investigated form a monophyletic cluster, which, together with *M. verecundum* 107^T^, *M. seminis* ARNO^T^, the five accipitrid-associated *Mycoplasma* species (*M. aquilae*, *M. paraquilae*, *M. haliaeeti*, *M*. *milvi*, and *M. razini*), and *M. nasistruthionis* 2F1A^T^, constitutes a clade that is phylogenetically positioned within the *M. synoviae* cluster of the Hominis group of genus *Mycoplasma* (Figure 2a).

All ISR sequences obtained from the ten investigated strains had a length of 262 nt and were identical, with the exception of strain BRA285, which exhibited a single nucleotide polymorphism. The ISR sequence of 48589B^T^ displayed the highest similarity to that of *M. aquilae* 1449^T^ (97.34%), *M. paraquilae* 654^T^ (96.96%), *M. razini* 005V^T^ (96.63%), *M. seminis* ARNO^T^ (93.89%), *M. haliaeeti* VS42A^T^ (93.41%), *M. milvi* Z331B^T^ (92.34%), and *M. verecundum* 107^T^ (91.67%), taking into account the full coverage of the ISR sequences compared.

All investigated strains possessed partial *rpoB* gene sequences of 1698 nt, with the closest sequence match identified for *M. haliaeeti* VS42A^T^ (93.29%), *M. milvi* Z331B^T^ (92.71%), *M. paraquilae* 654^T^ (92.65%), *M. aquilae* 1449^T^ (92.53%), *M. razini* 005V^T^ (92.05%), *M. seminis* 2200 (90.41%), and *M. verecundum* 107^T^ (88.46%). The partial *rpoB* sequences showed a high degree of similarity among the investigated strains, with pairwise sequence identities ranging from 98.88 to 99.88%. Despite exceeding the generally recommended threshold of 90–91% for species differentiation in mycoplasmas based on *rpoB* [43], these values were still below the thresholds recently proposed for distinguishing species within the *M. aquilae* complex, which comprises *M. aquilae*, *M. paraquilae*, *M. haliaeeti*, and *M. milvi* [20]. As observed for 16S rRNA gene sequences, the phylogenetic tree based on *rpoB* sequences illustrates a clear separation of the 48589B^T^ strain cluster from strains of related species (Figure 2b), which further supports the conclusion that the investigated strains represent a novel species within the *M. synoviae* cluster.

### 3.3. Phenotypic Traits and MALDI-ToF Mass Spectrometry Results

The examined strains neither metabolized glucose nor hydrolyzed arginine or urea. No hemolysis was observed on Columbia agar containing 5% sheep blood, and ‘film and spots’ production was absent in colonies grown on modified Hayflick’s agar for up to 10 days. Altogether, no distinguishing phenotypic characteristics were observed that differentiate the tested strains from their relatives.

**Figure 3:**
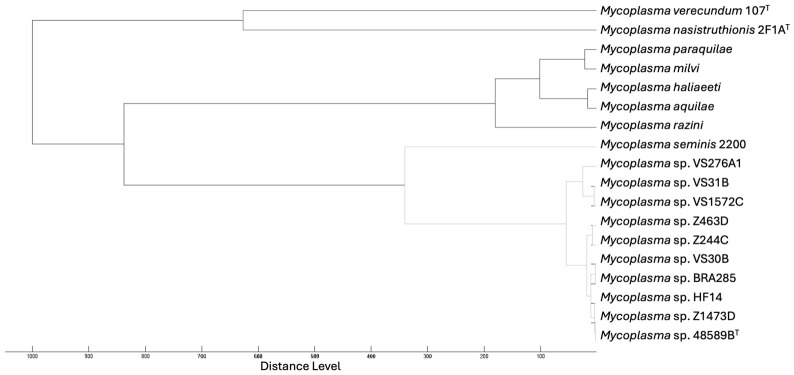
MALDI-ToF mass spectrometry score-oriented dendrogram based on distances between spectra from 48589B^T^ and related isolates and their closest relatives.

In MALDI-ToF mass spectrometric analysis, however, the ten strains formed a homogeneous cluster that was clearly distinct from the spectra generated for the phylogenetically closely related species *M. seminis*, *M. nasistruthionis*, *M. verecundum*, and the accipitrid-associated species *M. aquilae*, *M. paraquilae*, *M. haliaeeti*, *M. milvi*, and *M. razini* (Figure 3).

### 3.4. Genomic Traits and Coherence and Phylogenomics

The hybrid assembly combining Illumina short-read and Nanopore long-read data obtained from the proposed type strain 48589B^T^ yielded a circular chromosome with a sequencing coverage of 850.0×. The genome comprised 897,976 bp and exhibited a G+C content of 29.2%. Genome annotation of strain 48589B^T^ (CM135954) identified 687 predicted coding sequences (CDSs), of which 174 (25.3%) were classified as hypothetical proteins. The genome harbored three complete copies each of the 5S, 16S, and 23S rRNA genes. Whereas the 16S and 23S rRNA genes were organized into three operons, the 5S rRNA genes were located separately at more distant genomic positions. Comparison of the three 16S rRNA gene copies demonstrated only minor inter-operon sequence polymorphisms (a total of four base differences), similar to the minor differences observed when comparing the Sanger consensus sequences of the ten strains studied. Additional genomic characteristics of 48589B^T^ and of the remaining strains are summarized in Table 2.

The average nucleotide identity analyses performed between 48589B^T^ and closely related *Mycoplasma* species yielded ANIb values ranging from 76.45 to 80.58% (Table S1) and ANIm values between 85.56 and 86.65% (Table S2). These values were clearly below the proposed species boundary threshold of 95–96% [44]. In contrast, a comparison within the 48589B^T^ strain’s cluster resulted in markedly higher ANIb values of 96.87–98.25% (Table S3) and ANIm values of 97.81–98.46% (Table S4), supporting the assignment of the ten investigated strains to a single species. Similarly, tetranucleotide signature correlation analysis produced TETRA coefficients ranging from 0.894 to 0.924 in a pairwise comparison between strain 48589B^T^ and related *Mycoplasma* taxa (Table S5). These coefficients remained well below the ≥0.989 threshold proposed for genomes belonging to the same species [44]. By comparison, genomes within the 48589B^T^ strain group exhibited TETRA values consistent with species-level relatedness (Table S6). The results of ANIb, ANIm, and TETRA analyses were further corroborated by low digital DNA-DNA hybridization estimates obtained for strain 48589B^T^ and its closest relatives, including *M. haliaeeti* VS42A^T^ (24.5%), *M. milvi* Z331B^T^ (24.5%), *M. aquilae* 1449^T^ (24.4%), *M. razini* 005V^T^ (23.9%), and *M. paraquilae* 654^T^ (23.7%). Collectively, these findings strongly support the classification of strain 48589B^T^ as representing a novel species within the genus *Mycoplasma*.

**Figure 4:**
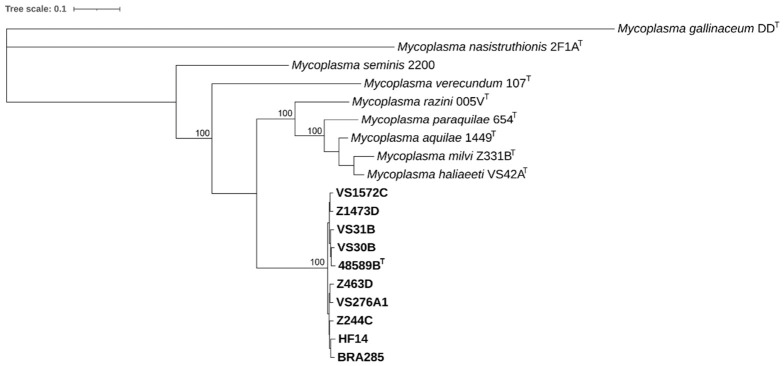
Phylogenomic tree derived from genome comparison of the ten strains investigated and their closest relatives within the *M. synoviae* cluster. *M. gallinaceum* DD^T^ was used as out-group organism. Numbers at nodes represent bootstrap confidence values (100 rounds). Tree scale, number of substitutions per site.

Phylogenomic reconstruction based on 273 single-copy coding genes, corresponding to an alignment of 101,344 amino acids and 304,032 nucleotides, yielded results consistent with those obtained from ANI/dDDH analyses and from phylogenetic studies of 16S rRNA genes and partial *rpoB* genes. The phylogenomic data therefore provide additional evidence that the investigated strains represent a separate species at the genomic level (Figure 4).

### 3.5. Functional and Comparative Genomics

Genome annotation indicated that the metabolic pathways of 48589B^T^ are highly similar to those described recently for related accipitrid-associated mycoplasmas [20]. In brief, the genome of 48589B^T^ lacks key enzymes of the upper Embden–Meyerhof–Parnas pathway and the oxidative pentose phosphate pathway, as well as a complete phosphoenolpyruvate phosphotransferase system. Conversely, genes for glycerol-3-phosphate metabolism and the lower EMP pathway were present, indicating glycerol-derived substrates as the primary entry point into central carbon metabolism. Downstream pathways for pyruvate oxidation and acetyl-CoA-to-acetate conversion appeared incomplete, suggesting restricted energy conservation from pyruvate.

Comparative genomic analysis identified multiple mobile genetic elements within the genomes analyzed in this study, highlighting their potential contribution to genomic plasticity. Insertion sequence (IS) family transposases were abundant in the circularized genome of 48589B^T^, whereas only a small number were detected in the draft genomes of the remaining strains (Table 2). This discrepancy is most likely attributable to differences in genome assembly quality, as repetitive elements such as IS sequences are known to be underrepresented or fragmented in draft assemblies derived from short-read sequencing data.

Additional mobile genetic elements included a ~14 kb putative prophage identified in strains 48589B^T^, Z1473D, Z244C (two copies), VS30B, VS276A1, and BRA285. This element displayed a gene content and overall organization reminiscent of the *M. arthritidis* MAV1 prophage group [45], although the encoded proteins shared relatively low amino acid identity (<40%) with MAV1 phage proteins. In contrast, a higher degree of similarity (58–87% amino acid identity) was observed with a related prophage identified in *M. paraquilae* 654^T^ (Figure S1). A second, larger prophage of approximately 33 kb was identified in strains VS31B, Z1473D, and HF14. Its gene content and structural organization resembled those of *M. agalactiae* MAgV1 and *M. molare* MAgV1-like prophages. Both gene synteny and protein sequences showed the highest similarity to an MAgV1-like prophage present in *M. milvi* Z331B^T^, with amino acid identities ranging from 63 to 86% among conserved core phage proteins (Figure S1).

In addition, mycoplasma integrative and conjugative elements (MICEs) were identified as single-copy insertions in the genomes of VS30B, Z244C, and Z1473D (Table 2) and were collectively designated ICETb_VS30B_. This element spanned approximately 29 kb and comprised 27 co-orientated genes, including a conserved set of backbone genes characteristic of MICEs [46]. A comparative analysis based on the amino acid identity of these backbone coding sequences indicated that ICETb_VS30B_ is related to MICEs described in accipitrid-associated *Mycoplasma* species, including *M. aquilae*, *M. paraquilae*, and *M. haliaeeti* [20]. The highest similarities (49–82% amino acid identity) were observed with backbone CDSs of the ICEP_aq654_ identified in *M. paraquilae* 654^T^ (Figure S2).

In silico screening for defense mechanisms against invading foreign DNA identified only restriction-modification systems (type I and II) in the genomes analyzed, whereas other systems such as CRISPR/Cas or toxin–antitoxin modules were not detected. The genomes also encoded several factors potentially associated with virulence and pathogenicity. These included proteins commonly described as cytadhesins in other *Mycoplasma* species (e.g., P60, P68, P80, OppA, and variable surface lipoproteins), as well as a pair of the *Mycoplasma* Ig binding (MIB)/*Mycoplasma* Ig protease (MIP) immune evasion system [47]. In addition, multiple orphan MIP family Ig-specific serine endopeptidases were identified. Together, these features indicate adaptation to a host-associated lifestyle and suggest that the investigated strains may harbor determinants that could contribute to opportunistic behavior.

## 4. Conclusions

Collectively, the phenotypic, phylogenetic, and genomic data presented here provide compelling evidence that the investigated strains constitute a novel species of the genus *Mycoplasma*. However, the results indicate that within this cluster of closely related *Mycoplasma* species, cultural characteristics, phenotypic profiles, and 16S rRNA gene sequences possess limited discriminatory power and should therefore be interpreted alongside MALDI-ToF mass spectrometry and genome-level analyses, which provide decisive evidence supporting the novelty of the species described here. The authors propose the name *Mycoplasma tracheobuteonis* sp. nov. The epithet *tracheobuteonis* was chosen to reflect the predominant isolation of these strains from the trachea of common buzzards (Table 3).

## Figures and Tables

**Table 1:** Strain metadata and corresponding accession numbers (acc. no.) for 16S rRNA gene, 16S–23S intergenic spacer region (ISR), and partial *rpoB* gene sequences.

Strain Designation	Host Species	Isolation Site	Year of Isolation	Country of Isolation	Acc. No. 16S rRNA	Acc. No. ISR	Acc. No. *rpoB*
48589B^T^	*Buteo buteo*	choana	2009	Germany	PZ033355	PZ036093	PZ044315
Z463D	*Buteo buteo*	trachea	2019	Austria	PZ033361	PZ036099	PZ044321
Z1473D	*Buteo buteo*	trachea	2019	Austria	PZ033362	PZ036100	PZ044322
VS1572C	*Buteo buteo*	trachea	2019	Austria	PZ033359	PZ036097	PZ044319
Z244C	*Buteo buteo*	trachea	2020	Austria	PZ033360	PZ036098	PZ044320
VS30B	*Buteo buteo*	trachea	2021	Austria	PZ033356	PZ036094	PZ044316
VS31B	*Buteo buteo*	trachea	2021	Austria	PZ033357	PZ036095	PZ044317
VS276A1	*Buteo buteo*	trachea	2022	Austria	PZ033358	PZ036096	PZ044318
HF14	*Aquila fasciata*	choana	2005	Spain	PZ033364	PZ036102	PZ044324
BRA285	*Haliaeetus albicilla*	choana	2009	Germany	PZ033363	PZ036101	PZ044323

**Table 2:** Genomic characteristics of the proposed type strain 48589B^T^ (finished genome) and its related strains Z463D, Z1473D, VS1572C, Z244C, VS30B, VS31B, VS276A1, HF14, and BRA285 (draft genomes).

	48589B^T^	Z463D	Z1473D	VS1572C	Z244C	VS30B	VS31B	VS276A1	HF14	BRA285
Accession number	CM135954	JBTKTC010000000	JBTKTB010000000	JBTKTE010000000	JBTKTD010000000	JBTKTH010000000	JBTKTG010000000	JBTKTF010000000	JBTKTI010000000	JBTKTJ01 0000000
Genome size (bp)	897,976	814,744	909,243	852,966	879,432	872,886	873,148	839,335	847,403	845,754
Genome coverage	850×	186×	203×	222×	197×	185×	200×	215×	210×	220×
Contig #	1	31	45	49	50	44	34	58	37	39
Contig N50 (kB)	-	46.4	35.2	42.2	33.2	35.8	59.1	35.8	42.2	32.2
G+C (%)	29.2	29.1	29.1	29.0	29.0	29.0	29.2	29.1	29.3	29.1
Genes (total)	744	646	747	682	712	700	696	689	691	669
CDSs (total)	701	609	711	648	676	664	660	651	653	633
CDSs (with protein)	687	607	709	646	674	662	658	647	649	628
Genes (RNA)	43	37	36	34	36	36	36	38	38	36
tRNAs	31	31	31	31	31	31	31	31	31	31
ncRNAs	3	3	3	2	3	3	3	3	3	3
Pseudo-genes	14	2	2	2	2	2	2	4	4	5
Transposases	32 (+5 pseudo) ^a^	4 ^b^	5 ^c^	9 ^d^	4 ^e^	6 ^f^	7 ^g^	8 ^h^	6 ^i^	6 ^j^
MICEs	-	-	ICETb_VS30B_	-	ICETb_VS30B_	ICETb_VS30B_	-	-	-	-
Prophages	MAV1-like	-	MAV1-like MAgV1-like	-	MAV1-like	MAV1-like	MAgV1-like	MAV1-like	MAgV1-like	MAV1-like

^a^ Five IS1634-, 11 IS256-(five of them pseudogenes), 15 IS30-, and one IS3-family transposases; five unclassified transposases. ^b^ One IS30-family transposase; three unclassified transposases. ^c^ One IS1634- and two IS30-family transposases; two unclassified transposases. ^d^ One IS1634- and six IS30-family transposases; two unclassified transposases. ^e^ One IS256- and one IS3-family transposases; two unclassified transposases. ^f^ Three IS30-family transposases; three unclassified transposases. ^g^ One IS1634-, three IS30- and one IS3-family transposases; two unclassified transposases. ^h^ One IS1634-, one IS256-, three IS30- and one IS3-family transposases; two unclassified transposases. ^i^ Two IS1634- and two IS30-family transposases; two unclassified transposases. ^j^ One IS1634-, one IS256-, three IS30-, and one IS3-family transposases.

**Table 3:** Description of *Mycoplasma tracheobuteonis* sp. nov.

Genus name	*Mycoplasma*
Species name	*Mycoplasma tracheobuteonis*
Species epithet	*tracheobuteonis*
Species status	sp. nov.
Species etymology	tra.che.o.bu.te.o’nis. Gr. fem. n. *tracheia*, windpipe; N.L. gen. n. *buteonis* of *Buteo*, a bird genus; N.L. gen. n. *tracheobuteonis*, of the trachea of a buzzard (*Buteo*), referring to the site of isolation and principal host
Description of the new taxon and diagnostic traits	Cells are wall-less and predominantly spherical to oval in morphology. Typical fried-egg colonies develop within 3 days of incubation on modified Hayflick’s agar at 37 °C in an atmosphere containing 5% CO_2_. Growth occurs between 20 °C and 42 °C, with optimal growth at 37 °C. Cells are non-motile. Neither arginine nor urea is hydrolyzed, and acid production from glucose is absent. MALDI-ToF mass spectrometry profiles are distinct from those of closely related *Mycoplasma* species. Although 16S rRNA gene and 16S–23S intergenic spacer region sequences show high similarity to related taxa, they allow differentiation from neighboring species. Partial *rpoB* gene sequences are unique and enable clear discrimination from closely related species. Genome similarity indices together with phylogenomic analyses further support the distinct species status of the investigated strains.
Country of origin	Germany
Region of origin	Saxony
Date of isolation	30 September 2009
Source of isolation	Choana of a common buzzard (*Buteo buteo*)
Sampling date	16 September 2009
Latitude	51°19′06.4′’ N
Longitude	12°23′28.2′’ E
Altitude (meters above sea level)	115
16S rRNA gene accession number	PZ033355
Genome accession number	CM135954
Genome status	Complete
Genome size (bp)	897,976
G + C (%)	29.2
Number of strains in study	10
Source of isolation of non-type strains	Z463D—trachea of a common buzzard (*Buteo buteo*), Austria (2019); Z1473D—trachea of a common buzzard (*Buteo buteo*), Austria (2019); VS1572C—trachea of a common buzzard (*Buteo buteo*)*,* Austria (2019); Z244C—trachea of a common buzzard (*Buteo buteo*), Austria (2020); VS30B—trachea of a common buzzard (*Buteo buteo*), Austria (2021); VS31B—trachea of a common buzzard (*Buteo buteo*), Austria (2021); VS276A1—trachea of a common buzzard (*Buteo buteo*), Austria (2022); HF14—choana of a Bonelli’s eagle (*Aquila fasciata*), Spain (2005); BRA285—choana of a white-tailed eagle (*Haliaeetus albicilla*), Germany (2009)
Designation of the type strain	48589B^T^
Strain collection numbers	DSM 115882^T^, NCTC 14927^T^

## Data Availability

Genome assemblies and marker gene sequences generated in this study have been deposited in the NCBI GenBank database. The corresponding accession numbers are provided in the main text, tables, and Supplementary Materials.

## Supplementary Materials

The following supporting information can be downloaded at: https://www.mdpi.com/article/10.3390/microorganisms14061224/s1. Table S1: Average nucleotide identities based on BLAST (ANIb, in %) between the genome of 48589B^T^ and those of closely related *Mycoplasma* species. Values in brackets represent aligned nucleotides in %. Values for ‘48589B^T^ versus strain of related species’ genome pairs are highlighted in blue. Table S2: Average nucleotide identities based on MUMmer (ANIm, in %) between the genome of 48589B^T^ and those of closely related *Mycoplasma* species. Values in brackets represent aligned nucleotides in %. Values for ‘48589B^T^ versus strain of related species’ genome pairs are highlighted in blue. Table S3: Average nucleotide identities based on BLAST (ANIb, in %) between genomes of 48589B^T^ and its related strains, and of closely related *M. razini* 005V^T^. Values in brackets represent aligned nucleotides in %. ANIb values above the proposed species delineation threshold of 95–96% are highlighted in bold. Table S4: Average nucleotide identities based on MUMmer (ANIm, in %) between genomes of 48589B^T^ and its related strains, and of closely related *M. razini* 005V^T^. Values in brackets represent aligned nucleotides in %. ANIb values above the proposed species delineation threshold of 95–96% are highlighted in bold. Table S5: Tetranucleotide signature correlation (TETRA) coefficients between the genome of 48589B^T^ and those of closely related *Mycoplasma* species. TETRA coefficients for ‘48589B^T^ versus strain of related species’ genome pairs are highlighted in bold. Table S6: Tetranucleotide signature correlation (TETRA) coefficients between genomes of 48589B^T^ and its related strains, and of closely related *M. razini* 005V^T^. TETRA coefficients above cutoff (≥0.999) or in range (≥0.989) are highlighted in bold and in green or blue, respectively. Figure S1: (a) A prophage identified in *M. tracheobuteonis* sp. nov. resembling φMAV1 of *M. arthritidis,* sharing only <40% aa identity to MAV1 phage proteins but showing higher similarity with a related prophage identified in *M. paraquilae* 654^T^. No homologues were identified for non-labelled proteins. GenBank locus tag numbers of the first and the last prophage proteins are indicated below the MAV1-like prophage of 48589B^T^. HtpN—HtpB, structural proteins; RepB, replicative DNA helicase, A and P, replication initiators; MarMP, putative C5 methylase; MarRP, transcriptional regulator; Int, integrase, Exis, excisionase, Vir, protein to exclude superinfecting phage; Imm, phage repressor; (**b**) A large prophage identified in *M. tracheobuteonis* sp. nov. reminiscent of *M. agalactiae* MAgV1-like prophages identified in *M. molare* H542^T^ and *M. milvi* Z331B^T^. GenBank locus tag numbers of the first and the last prophage proteins are indicated below the MAgV1-like prophage of VS31B. H, helicase; Pol, DNA polymerase; D, DNA primase; X, Xer recombinase; C, prohead protease; P, portal protein; T, terminase. Figure S2: Mycoplasma Integrative and Conjugative Element (MICE) backbone and structural organization of a MICE identified in the genome of VS30B (ICETb_VS30B_) related to ICEP_aq654_ identified in *M. paraquilae* 654^T^. MICE backbone CDS conserved across MICEs (labeled by *) or that may be absent or truncated in MICEs are represented by color filled arrows. GenBank locus tag numbers of the first and the last MICE genes are indicated below ICETb_VS30B_. SSBP: single-stranded DNA binding protein, P: pilin-like protein, ParA: ParA family protein, REase: restriction endonuclease, MTase: methyltransferase, IR: inverted repeats.
