# Supplementary material for: Mycoplasma tracheobuteonis sp. nov., a Novel Respiratory Mycoplasma Species from the Common Buzzard (Buteo buteo)
Source: Microorganisms. 2026 May 29;14(6):1224. doi: 10.3390/microorganisms14061224 (PMC13304146; doi:10.3390/microorganisms14061224)
Supplement: Supplementary file 1 [file microorganisms-14-01224-s001.zip › microorganisms-4313107-supplementary.pdf]

***Mycoplasma tracheobuteonis* sp. nov., a novel respiratory *Mycoplasma* species from the common buzzard (*Buteo buteo*)**

Sarah Kugler, Anna Kübber-Heiss, Nora Dinhopl, Angelika Auer, Igor Loncaric, Volker Schmidt, Ana S. Ramirez and Joachim Spergser

Supplementary Material:

Results and discussion

Genomic features, genomic coherence and phylogenomics

Table S1: Average nucleotide identities based on BLAST (ANIb, in %) between the genome of 48589B^T and those of closely related *Mycoplasma* species. Values in brackets represent aligned nucleotides in %. Values for '48589B^T versus strain of related species' genome pairs are highlighted in blue.

Strains (genome acc. no.)	1	2	3	4	5	6	7	8
(1) 48589B^T (CM135954)	*	**80.52** [62.03]	**79.80** [61.57]	**80.52** [61.98]	**80.58** [59.98]	**80.27** [59.17]	**77.39** [54.67]	**76.45** [57.12]
(2) *M. aquilae* 1449^T (CP182208)	**79.96** [61.20]	*	90.77 [75.75]	91.56 [75.95]	90.49 [69.71]	83.23 [64.07]	76.57 [53.79]	76.69 [52.50]
(3) *M. paraquilae* 654^T (CP182209)	**79.84** [60.89]	90.86 [79.20]	*	86.89 [77.51]	86.37 [73.72]	83.03 [68.92]	76.84 [52.97]	77.27 [52.80]
(4) *M. haliaeeti* VS42A^T (CP182210)	**80.38** [62.43]	91.81 [80.27]	86.80 [79.12]	*	93.03 [77.12]	83.29 [70.05]	76.72 [54.66]	76.93 [54.77]
(5) *M. milvi* Z331B^T (CP182211)	**80.45** [62.59]	90.97 [75.04]	86.38 [76.80]	93.04 [78.59]	*	83.10 [71.59]	77.04 [56.28]	77.20 [55.82]
(6) *M. razini* 005V^T (CP182212)	**80.36** [64.51]	83.09 [72.78]	82.97 [74.45]	83.29 [74.47]	82.80 [74.49]	*	77.53 [55.10]	77.42 [57.30]
(7) *M. seminis* 2200 (CP132191)	**77.36** [46.13]	77.18 [45.22]	77.20 [43.38]	76.92 [44.86]	77.49 [43.38]	77.76 [41.23]	*	76.67 [44.33]
(8) *M. verecundum* 107^T (CP137850)	**76.59** [52.60]	76.86 [51.24]	77.53 [48.71]	77.09 [50.28]	77.37 [49.35]	77.67 [48.35]	76.94 [51.09]	*

Table S2: Average nucleotide identities based on MUMmer (ANIm, in %) between the genome of 48589B^T and those of closely related *Mycoplasma* species. Values in brackets represent aligned nucleotides in %. Values for '48589B^T versus strain of related species' genome pairs are highlighted in blue.

Strains (genome acc. no.)	1	2	3	4	5	6	7	8
(1) 48589B^T (CM135954)	*	**86.57** [43.83]	**86.42** [38.71]	**86.53** [44.40]	**86.42** [43.04]	**86.65** [39.47]	**85.56** [28.97]	**85.60** [23.91]
(2) *M. aquilae* 1449^T (CP182208)	**86.57** [41.67]	*	92.19 [78.61]	93.74 [77.29]	92.69 [69.97]	87.82 [52.49]	85.69 [24.58]	85.47 [22.72]
(3) *M. paraquilae* 654^T (CP182209)	**86.40** [39.48]	92.19 [82.41]	*	89.28 [75.27]	88.84 [70.78]	87.36 [57.37]	85.55 [24.51]	85.40 [24.02]
(4) *M. haliaeeti* VS42A^T (CP182210)	**86.53** [44.89]	93.74 [81.36]	89.30 [75.25]	*	94.55 [77.96]	87.99 [57.63]	85.61 [27.40]	85.62 [23.81]
(5) *M. milvi* Z331B^T (CP182211)	**86.44** [45.22]	92.69 [77.54]	88.84 [74.18]	94.55 [81.39]	*	87.75 [59.13]	85.93 [26.22]	85.39 [26.83]
(6) *M. razini* 005V^T (CP182212)	**86.65** [43.05]	87.82 [60.12]	87.33 [62.12]	87.99 [62.11]	87.75 [61.03]	*	85.47 [29.69]	85.43 [28.00]
(7) *M. seminis* 2200 (CP132191)	**85.56** [24.76]	85.69 [21.78]	85.55 [20.49]	85.61 [22.92]	85.93 [20.90]	85.47 [22.97]	*	85.16 [19.32]
(8) *M. verecundum* 107^T (CP137850)	**85.57** [22.90]	85.47 [22.59]	85.40 [22.47]	85.62 [22.29]	85.39 [23.99]	85.43 [24.38]	85.16 [21.71]	*

Table S3: Average nucleotide identities based on BLAST (ANIb, in %) between genomes of 48589B^T and its related strains, and of closely related *M. razini* 005V^T. Values in brackets represent aligned nucleotides in %. ANIb values above the proposed species delineation threshold of 95–96% are highlighted in bold.

Strains<br>(genome acc. no.)	1	2	3	4	5	6	7	8	9	10	11
(1) 48589B^T<br>(CM135954)	 *	**97.79**<br>[82.45]	**97.69**<br>[83.99]	**97.65**<br>[82.20]	**97.68**<br>[82.47]	**98.08**<br>[85.56]	**97.91**<br>[83.87]	**97.54**<br>[82.61]	**96.87**<br>[81.11]	**97.60**<br>[83.75]	80.27<br>[59.17]
(2) Z463D<br>(JBTKTC010000000)	**97.78**<br>[90.44]	 *	**97.89**<br>[89.49]	**97.64**<br>[89.04]	**97.66**<br>[89.02]	**97.96**<br>[90.96]	**98.01**<br>[91.45]	**97.89**<br>[89.60]	**97.12**<br>[87.55]	**97.65**<br>**[90.19]**	79.67<br>[63.68]
(3) Z1473D<br>(JBTKTB010000000)	**97.67**<br>[83.50]	**97.90**<br>[80.63]	 *	**97.91**<br>[84.55]	**97.50**<br>[85.02]	**97.95**<br>[86.93]	**97.88**<br>[86.68]	**97.95**<br>[82.34]	**97.03**<br>[83.21]	**97.64**<br>[84.11]	80.10<br>[57.78]
(4) VS1572C<br>(JBTKTE010000000)	**97.77**<br>[85.96]	**97.62**<br>[85.36]	**98.03**<br>[89.94]	 *	**97.83**<br>[88.38]	**97.87**<br>[88.79]	**98.13**<br>[87.35]	**98.21**<br>[86.63]	**97.28**<br>[83.96]	**97.82**<br>[85.53]	79.78<br>[61.17]
(5) Z244C<br>(JBTKTD010000000)	**97.65**<br>[85.69]	**97.66**<br>[82.62]	**97.58**<br>[88.20]	**97.67**<br>[85.51]	 *	**97.84**<br>[88.03]	**97.74**<br>[83.51]	**97.71**<br>[85.42]	**97.29**<br>[82.82]	**97.61**<br>[86.68]	79.80<br>[60.25]
(6) VS30B<br>(JBTKTH010000000)	**98.18**<br>[87.61]	**97.96**<br>[85.07]	**97.99**<br>[88.85]	**97.85**<br>[85.79]	**97.64**<br>[87.63]	 *	**98.25**<br>[86.48]	**97.64**<br>[84.76]	**96.76**<br>[82.34]	**97.51**<br>[86.59]	79.68<br>[60.41]
(7) VS31B<br>(JBTKTG010000000)	**97.99**<br>[85.33]	**97.80**<br>[85.05]	**97.83**<br>[89.84]	**97.96**<br>[85.31]	**97.58**<br>[84.31]	**98.20**<br>[86.38]	 *	**97.96**<br>[83.49]	**96.92**<br>[85.30]	**97.37**<br>[84.01]	79.74<br>[59.34]
(8) VS276A1<br>(JBTKTF010000000)	**97.62**<br>[88.84]	**97.91**<br>[87.06]	**98.07**<br>[89.63]	**98.13**<br>[88.03]	**97.83**<br>[88.99]	**97.71**<br>[88.77]	**98.09**<br>[88.38]	 *	**97.38**<br>[86.20]	**97.74**<br>[89.32]	79.95<br>[62.04]
(9) HF14<br>(JBTKTI010000000)	**97.10**<br>[84.48]	**97.18**<br>[83.40]	**97.24**<br>[87.87]	**97.43**<br>[83.36]	**97.41**<br>[85.07]	**97.05**<br>[83.37]	**97.18**<br>[87.20]	**97.43**<br>[84.29]	 *	**97.55**<br>[84.97]	79.81<br>[60.15]
(10) BRA285<br>(JBTKTJ010000000)	**97.91**<br>[88.21]	**97.78**<br>[86.51]	**97.90**<br>[88.69]	**97.82**<br>[85.98]	**97.66**<br>[88.15]	**97.79**<br>[88.53]	**97.73**<br>[86.07]	**97.85**<br>[87.91]	**97.56**<br>**[85.80]**	 *	79.95<br>[61.64]
(11) *M. razini* 005V^T<br>(CP182212)	80.36<br>[64.51]	79.93<br>[62.90]	79.92<br>[62.38]	79.93<br>[62.74]	80.09<br>[63.06]	79.98<br>[62.83]	79.84<br>[62.75]	80.00<br>[62.39]	79.97<br>[62.32]	80.10<br>[63.08]	 *

Table S4: Average nucleotide identities based on MUMmer (ANIm, in %) between genomes of 48589B^T and its related strains, and of closely related *M. razini* 005V^T. Values in brackets represent aligned nucleotides in %. ANIb values above the proposed species delineation threshold of 95–96% are highlighted in bold.

Strains (genome acc. no.)	1	2	3	4	5	6	7	8	9	10	11
(1) 48589B^T		**98.20**	**98.19**	**98.22**	**98.23**	**98.31**	**98.23**	**98.24**	**97.93**	**98.18**	86.65
(CM135954)	*	[90.12]	[92.11]	[89.52]	[90.26]	[93.69]	[92.08]	[90.28]	[87.18]	[90.87]	[39.47]
(2) Z463D	**98.20**		**98.32**	**98.22**	**98.21**	**98.37**	**98.35**	**98.23**	**97.83**	**98.05**	86.05
(JBTKTC010000000)	[96.51]	*	[95.59]	[94.59]	[95.10]	[96.76]	[97.04]	[95.85]	[92.68]	[95.88]	[42.47]
(3) Z1473D	**98.19**	**98.33**		**98.31**	**98.33**	**98.37**	**98.24**	**98.39**	**97.93**	**98.18**	86.03
(JBTKTB010000000)	[88.50]	[85.84]	*	[90.31]	[90.35]	[92.37]	[91.97]	[88.50]	[87.17]	[88.81]	[38.60]
(4) VS1572C	**98.22**	**98.22**	**98.31**		**98.39**	**98.32**	**98.40**	**98.46**	**98.01**	**98.21**	86.19
(JBTKTE010000000)	[91.63]	[90.36]	[96.12]	*	[94.27]	[95.04]	[93.79]	[92.95]	[89.52]	[91.57]	[40.13]
(5) Z244C	**98.24**	**98.21**	**98.33**	**98.39**		**98.32**	**98.40**	**98.39**	**98.05**	**98.20**	86.01
(JBTKTD010000000)	[91.33]	[87.94]	[94.50]	[91.23]	*	[95.02]	[89.00]	[91.33]	[87.03]	[91.95]	[40.78]
(6) VS30B	**98.31**	**98.37**	**98.37**	**98.32**	**98.32**		**98.37**	**98.30**	**97.81**	**98.08**	86.12
(JBTKTH010000000)	[93.78]	[90.18]	[95.77]	[92.58]	[94.17]	*	[92.99]	[90.63]	[86.22]	[91.54]	[39.52]
(7) VS31B	**98.22**	**98.35**	**98.23**	**98.40**	**98.40**	**98.37**		**98.46**	**97.92**	**98.09**	86.07
(JBTKTG010000000)	[91.85]	[90.64]	[95.69]	[91.54]	[90.10]	[93.28]	*	[90.45]	[90.25]	[89.25]	[39.40]
(8) VS276A1	**98.24**	**98.23**	**98.39**	**98.46**	**98.39**	**98.30**	**98.46**		**98.01**	**98.15**	86.01
(JBTKTF010000000)	[93.88]	[93.11]	[95.76]	[94.44]	[94.63]	[94.23]	[94.02]	*	[91.40]	[95.61]	[42.27]
(9) HF14	**97.93**	**97.83**	**97.93**	**98.01**	**98.05**	**97.81**	**97.92**	**98.01**		**98.14**	86.04
(JBTKTI010000000)	[89.41]	[88.89]	[93.34]	[89.79]	[90.35]	[88.71]	[92.87]	[90.45]	*	[90.72]	[41.54]
(10) BRA285	**98.18**	**98.05**	**98.18**	**98.21**	**98.20**	**98.08**	**98.09**	**98.15**	**98.14**		86.04
(JBTKTJ010000000)	[93.99]	[92.32]	[95.22]	[92.16]	[94.05]	[94.56]	[91.95]	[94.68]	[91.05]	*	[42.14]
(11) *M. razini* 005V^T	86.65	86.05	86.03	86.19	86.01	86.12	86.07	86.01	86.06	86.04	
(CP182212)	[43.05]	[42.05]	[42.64]	[41.62]	[43.58]	[41.95]	[41.82]	[43.09]	[42.73]	[43.62]	*

Table S5: Tetranucleotide signature correlation (TETRA) coefficients between the genome of 48589B^T and those of closely related *Mycoplasma* species. TETRA coefficients for '48589B^T versus strain of related species' genome pairs are highlighted in bold.

Strains (genome acc. no.)	1	2	3	4	5	6	7	8
(1) 48589B^T (CM135954)	*	**0.916**	**0.924**	**0.894**	**0.908**	**0.918**	**0.907**	**0.912**
(2) *M. aquilae* 1449^T (CP182208)	**0.916**	*	0.983	0.986	0.988	0.971	0.880	0.904
(3) *M. paraquilae* 654^T (CP182209)	**0.924**	0.983	*	0.963	0.979	0.979	0.880	0.932
(4) *M. haliaeeti* VS42A^T (CP182210)	**0.894**	0.986	0.963	*	0.989	0.965	0.870	0.882
(5) *M. milvi* Z331B^T (CP182211)	**0.908**	0.988	0.979	0.989	*	0.977	0.886	0.903
(6) *M. razini* 005V^T (CP182212)	**0.918**	0.971	0.979	0.965	0.977	*	0.874	0.909
(7) *M. seminis* 2200 (CP132191)	**0.907**	0.880	0.880	0.870	0.886	0.874	*	0.907
(8) *M. verecundum* 107^T (CP137850)	**0.912**	0.904	0.932	0.882	0.903	0.909	0.907	*

Table S6: Tetranucleotide signature correlation (TETRA) coefficients between genomes of 48589B^{T} and its related strains, and of closely related *M. razini* 005V^{T}. TETRA coefficients above cutoff (≥0.999) or in range (≥0.989) are highlighted in bold and in green or blue, respectively.

Strains (genome acc. no.)	1	2	3	4	5	6	7	8	9	10	11
(1) 48589B^{T} (CM135954)	*	**0.996**	**0.989**	**0.996**	**0.996**	**0.996**	**0.994**	**0.996**	**0.993**	**0.996**	0.918
(2) Z463D (JBTKTC010000000)	**0.996**	*	**0.989**	**0.997**	**0.995**	**0.996**	**0.993**	**0.999**	**0.993**	**0.997**	0.923
(3) Z1473D (JBTKTB010000000)	**0.989**	**0.989**	*	**0.993**	**0.995**	**0.995**	**0.997**	**0.992**	**0.997**	**0.992**	0.896
(4) VS1572C (JBTKTE010000000)	**0.996**	**0.997**	**0.993**	*	**0.997**	**0.998**	**0.996**	**0.998**	**0.995**	**0.997**	0.920
(5) Z244C (JBTKTD010000000)	**0.996**	**0.995**	**0.995**	**0.997**	*	**0.998**	**0.997**	**0.997**	**0.996**	**0.997**	0.912
(6) VS30B (JBTKTH010000000)	**0.996**	**0.996**	**0.995**	**0.998**	**0.998**	*	**0.997**	**0.998**	**0.996**	**0.998**	0.917
(7) VS31B (JBTKTG010000000)	**0.994**	**0.993**	**0.997**	**0.996**	**0.997**	**0.997**	*	**0.995**	**0.998**	**0.996**	0.905
(8) VS276A1 (JBTKTF010000000)	**0.996**	**0.999**	**0.992**	**0.998**	**0.997**	**0.998**	**0.995**	*	**0.995**	**0.998**	0.921
(9) HF14 (JBTKTI010000000)	**0.993**	**0.993**	**0.997**	**0.995**	**0.996**	**0.996**	**0.998**	**0.995**	*	**0.995**	0.905
(10) BRA285 (JBTKTJ010000000)	**0.996**	**0.997**	**0.992**	**0.997**	**0.997**	**0.998**	**0.996**	**0.998**	**0.995**	*	0.920
(11) *M. razini* 005V^{T} (CP182212)	0.918	0.923	0.896	0.920	0.912	0.917	0.905	0.921	0.905	0.920	*

Functional and comparative genomics

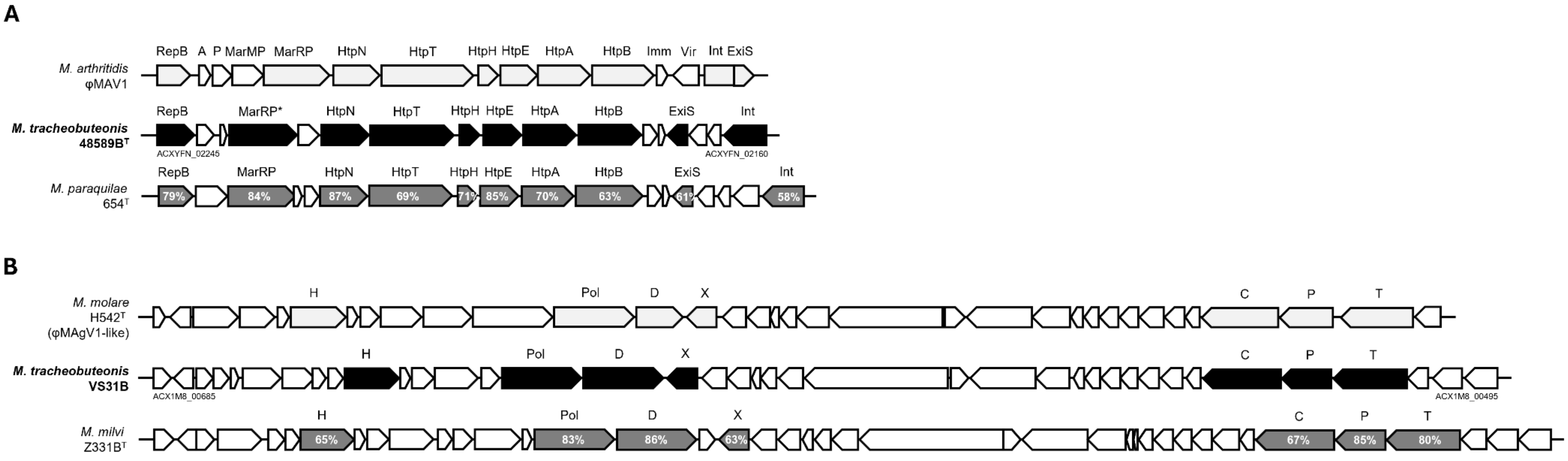

Fig S1: (A) A prophage identified in *M. tracheobuteonis* sp. nov. resembling φMAV1 of *M. arthritidis,* sharing only < 40% aa identity to MAV1 phage proteins but showing higher similarity with a related prophage identified in *M. paraquilae* 654^{T}. No homologues were identified for non-labelled proteins. GenBank locus tag numbers of the first and the last prophage proteins are indicated below the MAV1-like prophage of 48589B^{T}. HtpN - HtpB, structural proteins; RepB, replicative DNA helicase, A and P, replication initiators; MarMP, putative C5 methylase; MarRP, transcriptional regulator; Int, integrase, Exis, excisionase, Vir, protein to exclude superinfecting phage; Imm, phage repressor. (B) A large prophage identified in *M. tracheobuteonis* sp. nov. reminiscent of *M. agalactiae* MAgV1-like prophages identified in *M. molare* H542^{T} and *M. milvi* Z331B^{T}. GenBank locus tag numbers of the first and the last prophage proteins are indicated below the MAgV1-like prophage of VS31B. H, helicase; Pol, DNA polymerase; D, DNA primase; X, Xer recombinase; C, prohead protease; P, portal protein; T, terminase

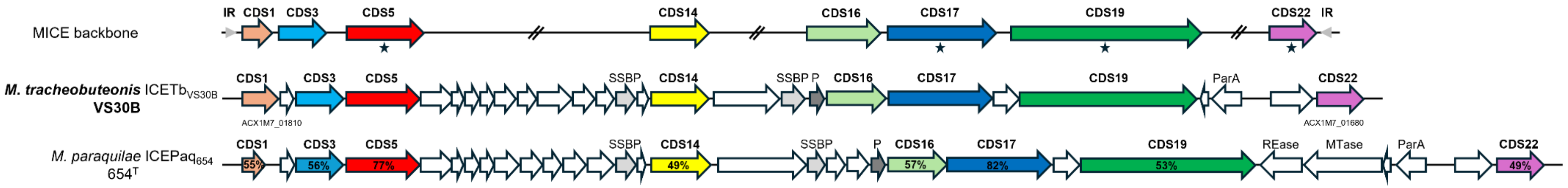

Fig. S2: Mycoplasma Integrative and Conjugative Element (MICE) backbone and structural organization of a MICE identified in the genome of VS30B (ICETb$_{VS30B}$) related to ICEP$_{aq654}$ identified in *M. paraquilae* 654^{T}. MICE backbone CDS conserved across MICEs (labeled by *) or that may be absent or truncated in MICEs are represented by color filled arrows. GenBank locus tag numbers of the first and the last MICE genes are indicated below ICETb$_{VS30B}$. SSBP: single-stranded DNA binding protein, P: pilin-like protein, ParA: ParA family protein, REase: restriction endonuclease, MTase: methyltransferase, IR: inverted repeats.
